# Exploring variation in ambulance calls and conveyance rates for adults with diabetes mellitus who contact the Northern Ireland Ambulance Service: a retrospective database analysis

**DOI:** 10.29045/14784726.2021.12.6.3.15

**Published:** 2021-12-01

**Authors:** Aoife Watson, Benjamin Clubbs Coldron, Benjamin Wingfield, Nigel Ruddell, Chris Clarke, Siobhan Masterson, Donna McConnell, Vivien Coates

**Affiliations:** Ulster University; University of Highlands and Islands; Clinical Translational Research and Innovation Centre; Northern Ireland Ambulance Service; Northern Ireland Ambulance Service; National Ambulance Service; Ulster University; Ulster University

**Keywords:** diabetes mellitus, emergency medical services

## Abstract

**Background:**

People with diabetes frequently contact the ambulance service about acute problems. Overall, treating diabetes and its associated complications costs the NHS 10% of the annual budget. Reducing unnecessary hospital admissions and ambulance attendances is a high priority policy for the NHS across the UK. This study aimed to determine the characteristics of emergency calls for people with diabetes who contact the ambulance service and are subsequently conveyed to hospital by the Northern Ireland Ambulance Service (NIAS).

**Methods:**

A retrospective dataset from the NIAS was obtained from the NIAS Trust’s Command and Control system relating to calls where the final complaint group was ‘Diabetes’ for the period 1 January 2017 to 23 November 2019.

**Results:**

Of a total 11,396 calls related to diabetes, 63.2% of callers to the NIAS were conveyed to hospital. Over half of the calls related to males, with 35.5% of callers aged 60–79. The more deprived areas had a higher frequency of calls and conveyance to hospital, with this decreasing as deprivation decreased. Calls were evenly distributed across the week, with the majority of calls originating outside of GP working hours, although callers were more likely to be conveyed to hospital during working hours. Calls from healthcare professionals were significantly more likely to be conveyed to hospital, despite accounting for the minority of calls.

**Conclusion:**

This research found that older males were more likely to contact the ambulance service but older females were more likely to be conveyed to hospital. The likelihood of conveyance increased if the call originated from an HCP or occurred during GP working hours. The availability of alternative care pathways has the potential to reduce conveyance to hospital, which has been particularly important during the COVID-19 pandemic. Integration of data is vitally important to produce high quality research and improve policy and practice in this area.

## Introduction

Diabetes is a condition characterised by chronic hyperglycaemia or high blood glucose levels (Punthakee et al., 2018). This is caused by defective or inactive insulin production and secretion or insulin resistance, and results in a range of metabolic disorders (Goldenberg & Punthakee, 2013). In 2019, over 463 million people worldwide were living with diabetes, accounting for over 9% of the world’s population, with this prevalence rising (Saeedi et al., 2019). There are 3.9 million people in the UK diagnosed with diabetes, and over half a million estimated undiagnosed cases, with 99,833 of these individuals in Northern Ireland (Diabetes UK, 2019).

People with diabetes may experience acute complications such as hypoglycaemia or hyperglycaemia, sometimes resulting in emergencies such as diabetic ketoacidosis (DKA) or hyperglycaemic hyperosmolar non-ketotic coma. People with these acute complications often require ambulance assistance. Overall, treatment for diabetes and its associated complications costs the NHS 10% of the annual budget (Diabetes UK, 2014). Severe hypoglycaemia is an episode that requires assistance from another person to increase blood glucose levels (Chow & Seaquist, 2020). Severe hypoglycaemic events account for 48,000–98,400 emergency ambulance calls within the UK annually, with 63–73% of people who call safely left at home (Duncan & Fitzpatrick, 2016; Duncan et al., 2018). Reducing unnecessary hospital admissions and use of emergency services is a policy of high priority for the health services across the UK, with the aim to treat people as close to home as possible (NHS England, 2014).

The accessibility of healthcare services varies across regions and by time of day. Conveyance rates are associated with the accessibility of services by ambulance staff (Coles, 2018). The availability of services outside of normal GP working hours, 08:00 to 18:00 Monday to Friday, is reduced, influencing the conveyance of patients to hospital following ambulance call-outs. Any time outside of GP working hours is classified as out-of-hours. The scope of practice for paramedics and Emergency Medical Technicians (EMTs) in the Northern Ireland Ambulance Services (NIAS) varies, with EMTs being able to provide fewer treatment options than paramedics and paramedics being able to leave people at home, where appropriate, which may influence time spent on scene and conveyance rates (Pre-Hospital Emergency Care Council, 2018). Previous research has shown that when a paramedic is in attendance, conveyance to hospital is reduced (Van Woerden et al., 2020).

Economic and social factors that can influence health include income, education, occupation and inequalities due to race and ethnicity, with relationships shown between health and deprivation (Institute of Medicine & National Research Council, 2013). Highly deprived areas have been associated with poorer health status, increased incidence of chronic conditions and more multi-morbidities (Barnett et al., 2012; Dalstra et al., 2005; Peconi et al., 2019). As a result of this, there is frequently higher usage of primary care and emergency services in deprived areas (Majeed et al., 2000).

The aim of this study was to determine the characterisations of people who contact the NIAS for diabetes-related complications and which factors influence conveyance to hospital. To achieve this, the following objectives were formulated:

1. To examine associations between call volume and time of day.
2. To investigate if locality affects calls.
3. To determine whether these factors influence conveyance to hospital for further treatment.

We hypothesised that there would be a higher rate of calls and conveyance in more deprived areas and in areas where there is a longer drive time to hospital, and that there would be more out-of-hours callers conveyed to hospital. We posited that the findings might also show that more time is spent at scene on calls with a longer drive time and that calls made by healthcare professionals were more likely to result in patients being conveyed to hospital compared with other calls.

## Methods

### Study setting

The NIAS covers the whole of Northern Ireland, freely providing urgent, emergency and primary care services. They respond to emergency 999 calls and also provide non-urgent transportation for vulnerable members of the community. Northern Ireland has a population of around 1.9 million, with over 218,000 NIAS calls received in 2018–2019, an increase in calls from previous years (Northern Ireland Ambulance Service Trust, 2020). Approximately 37% of the Northern Ireland population live in rural areas. The NIAS is responsible for both urban and rural populations, including some of the most deprived localities in the UK (Department of Agriculture and Rural Development, 2016).

### Study design

A retrospective dataset from the NIAS was used to determine which patients were most likely to contact the ambulance service and subsequently be conveyed to hospital. Data were extracted from the NIAS Trust’s Command and Control system which records emergency call activity. The NIAS provided the data from 1 January 2017 to 23 November 2019 where the chief complaint was ‘Diabetes’. Data prior to 1 January 2017 had already been ‘flattened’ into a single table and so could not be extracted. Emergency calls made by the public are received by Emergency Medical Dispatchers, who use bespoke AMPDS (Advanced Medical Priority Dispatch System) software systems to categorise calls based on their urgency as life-threatening (Category A), serious but not life-threatening (Category B) or not serious or life-threatening (Category C) (Northern Ireland Ambulance Service, n.d.). Emergency Medical Dispatchers provide medical advice prior to the ambulance arriving and gather and record data including those outlined in Table 1.

**Table 1.** Data received from the Northern Ireland Ambulance Service.

Data received
Diagnostic code group = DIABETES
Call number
Patient age
Sex
Postcode
Call type
Call category
Method of call
Dispatch code/description
Date and time of call
Time of arrival and departure from scene
Time of resource clear
Conveyed to hospital
Hospital attended to by ambulance
Call stopped reason
Clear delay reason
Symptoms
Electoral ward
Super council district areas
Performance: best response time

Clinical data were stored on a separate system known as FORMIC that contained patient information. These data were not made available to us due to various constraints regarding accessing personal patient-identifiable data, as the ‘call-out’ and the clinical systems are not integrated with each other.

### Data manipulation

Based on the data received, we calculated the length of time spent at scene, the drive time and the length of time the resource was under use. Deprivation rankings were not provided but were calculated based on Super Output Areas (geographical areas characterised by NISRA to improve small area statistics reporting) that were determined from pick-up postcodes. This allowed Multiple Deprivation Measures (MDMs) to be mapped to the postcodes, which is the gold standard measure for estimating deprivation in NI (NISRA, 2017). MDMs were then grouped into MDM deciles from 1 to 10, with 1 being the most deprived areas and 10 being the least deprived.

Rurality was calculated based on distance between pick-up postcode and hospital, and drive time calculated from the pick-up postcode to the hospital. The distances were calculated using the Google distance matrix API (Application Programming Interface), which provides distance and travel time between two points, based on the road route recommended by Google (Google Developers, 2021).

### Data analysis

Descriptive and inferential statistics were carried out using SPSS version 25.0 and R version 3.6.3 (IBM, n.d.; R Project, n.d.). Statistical analysis included calculating frequencies, means with standard deviation (SD), odds ratios with 95% confidence intervals, Pearson’s correlation (r) and p values. Odds ratios were calculated using binary logistic regression analysis, with p < 0.05 classed as statistically significant (LoBiondo-Wood & Haber, 2014). This analysis included the following variables: age and sex; day of the week; time the call was made and method of call; and whether this impacted conveyance rates to hospital. Analysis on whether conveyance rates were affected by length of time spent at scene, drive time to hospital, rurality and deprivation ranking was also carried out, along with whether drive time influenced length of time spent at scene.

### Missing data

Any missing data were labelled as unknown in the dataset and included in the analysis.

### Patient and public involvement

No patient involvement.

## Results

The dataset contained 12,358 diabetes-related call-outs over the 35 months. Calls that pertained to children (<18 years old), cancelled calls, hoax calls, duplicate calls and calls where there was no patient found at the scene were excluded, leaving 11,396 records for analysis. An overview of the results is shown in Tables 2 and 3. Analysis of the data showed that in 2017, 2018 and 2019 there were respectively 3971, 3869 and 3556 diabetes-related calls made to the ambulance service, showing that the volume of calls remained relatively stable, although there was only 11 months of calls for 2019.

**Table 2.** Characteristics of calls and conveyance.

Risk factor	Levels	Conveyed to hospital		
		Yes	No	Yes/no combined
**Sex**	Male	3848 (61.3%)	2426 (38.7%)	6274 (55.1%)
	Female	3345 (65.7%)	1749 (34.3%)	5094 (44.7%)
	Unknown	8 (28.6%)	20 (71.4%)	28 (0.2%)
**Age groups**	18–39	1141 (55.7%)	906 (44.3%)	2047 (18.0%)
	40–59	1748 (57.9%)	1273 (42.1%)	3021 (26.5%)
	60–79	2635 (65.0%)	1416 (35%)	4051 (35.5%)
	80+	1627 (75.7%)	521 (24.3%)	2148 (18.8%)
	Unknown	50 (38.8%)	79 (61.2%)	129 (1.1%)
**MDM decile**	1, most deprived	1087 (61.9%)	669 (38.1%)	1756 (15.3%)
	2	940 (62.4%)	567 (37.6%)	1507 (13.2%)
	3	827 (63.2%)	482 (36.8%)	1309 (11.4%)
	4	798 (63.1%)	466 (36.9%)	1264 (11.0%)
	5	799 (68.5%)	368 (31.5%)	1167 (10.2%)
	6	599 (65.5%)	316 (34.5%)	915 (8.0%)
	7	697 (64.7%)	381 (35.3%)	1078 (9.4%)
	8	540 (65.1%)	290 (34.9%)	830 (7.3%)
	9	504 (58.5%)	358 (41.5%)	862 (7.5%)
	10, least deprived	456 (62.6%)	272 (37.4%)	728 (6.4%)
**Day of week**	Monday	1072 (64.0%)	603 (36.0%)	1675 (14.7%)
	Tuesday	1067 (64.5%)	586 (35.5%)	1653 (14.5%)
	Wednesday	994 (62.6%)	593 (37.4%)	1587 (13.9%)
	Thursday	983 (62.4%)	592 (37.6%)	1575 (13.8%)
	Friday	987 (63.6%)	565 (36.4%)	1552 (13.6%)
	Saturday	1038 (63.0%)	609 (37.0%)	1647 (14.5%)
	Sunday	1060 (62.1%)	647 (37.9%)	1707 (15.0%)
**In-/out-of-hours**	Working hours	3145 (68.0%)	1478 (32.0%)	4623 (40.6%)
	Out-of-hours	4056 (59.9%)	2717 (40.1%)	6773 (59.4%)
**Caller**	HCP	2428 (90.6%)	252 (9.4%)	2680 (23.5%)
	Non-HCP	4773 (54.8%)	3943 (45.2%)	8716 (76.5%)
**Time of call block**	00:00–06:00	795 (54.2%)	672 (45.8%)	1467 (12.9%)
	06:00–12:00	1858 (65.6%)	974 (34.4%)	2832 (24.9%)
	12:00–18:00	2719 (67.7%)	1300 (32.3%)	4019 (35.3%)
	18:00–00:00	1829 (59.4%)	1249 (40.6%)	3078 (27.0%)
**Drive time**	<15 minutes	3579 (100%)	N/A	3579 (31.4%)
	15–30 minutes	2632 (100%)	N/A	2632 (23.1%)
	30–45 minutes	840 (100%)	N/A	840 (7.4%)
	45–60 minutes	115 (100%)	N/A	115 (1.0%)
	>60 minutes	27 (100%)	N/A	27 (0.2%)
	Unknown	8 (0.2%)	N/A	4203 (36.9%)
**Time spent on scene**	<15 minutes	878 (99.7%)	3 (0.3%)	881 (7.7%)
	15–30 minutes	3269 (100%)	1 (0%)	3270 (28.7%)
	30–45 minutes	1881 (99.9%)	1 (0.1%)	1882 (16.5%)
	45–60 minutes	673 (100%)	0 (0%)	673 (5.9%)
	>60 minutes	354 (100%)	0 (0%)	354 (3.1%)
	Unknown	146 (3.4%)	4190 (96.6%)	4336 (38.9%)

**Table 3.** Binary regression analysis of risk factors associated with hospital conveyance.

Risk factor	Levels	OR (95% CI)	P value
**Sex**	Male	REF	REF
	Female	1.21 [1.12;1.30]	<0.01
**Age groups**	Continuous	1.35 [1.30;1.40]	<0.01
	18–39	REF	REF
	40–59	1.09 [0.98;1.22]	0.13
	60–79	1.49 [1.34;1.66]	<0.01
	80+	2.53 [2.22;2.89]	<0.1
**MDM decile**	Continuous	1.01 [0.00;1.02]	0.50
	1	REF	REF
	2	1.19 [1.03;1.37]	0.02
	3	0.98 [0.84;1.14]	0.78
	4	0.98 [0.84;1.14]	0.78
	5	1.15 [0.99;1.34]	0.07
	6	1.16 [0.98;1.36]	0.09
	7	1.01 [0.86;1.18]	0.95
	8	1.01 [0.85;1.20]	0.92
	9	1.10 [0.92;1.30]	0.30
	10	1.16 [0.97;1.39]	0.11
**Day of week**	Monday	1.02 [0.88;1.17]	0.811
	Tuesday	1.04 [0.90;1.20]	0.574
	Wednesday	0.96 [0.83;1.11]	0.577
	Thursday	0.95 [0.82;1.08]	0.494
	Friday	REF	REF
	Saturday	0.98 [0.84;1.14]	0.738
	Sunday	0.94 [0.81;1.08]	0.377
**In-/out-of-hours**	Working hours	1.43 [1.32;1.54]	<0.01
	Out-of-hours	REF	REF
**Caller**	HCP	7.95 [6.95;9.14]	<0.01
	Non-HCP	REF	REF
**Time of call block**	00:00–06:00	REF	REF
	06:00–12:00	1.61 [1.42;1.83]	<0.01
	12:00–18:00	1.77 [1.57;2.0]	<0.01
	18:00–00:00	1.24 [1.09;1.40]	<0.01

There was minimal variance in calls across the week, with Friday accounting for the lowest number of calls (13.6%) and Sunday the highest (15.0%). There was no difference between the likelihood of conveyance to hospital on a Tuesday than on a Friday (OR1.04, p = 0.57) or on a Sunday (OR0.94, p = 0.81).

The data showed that significantly more patients were conveyed to hospital than were left at home (63.2% vs. 36.8%; p < 0.01). Just over half of calls to the ambulance service related to male patients (55.1%), with calls regarding female patients making up 44.7%. Female patients were more likely to be conveyed to hospital than male patients (OR1.2, p < 0.01).

Patient ages ranged from 18 to 103 years old, with the mean age 60.2 years (SD = 19.6). Patients aged 60–79 years made up 35.5% calls, with 18–39-year-olds making up the lowest number of calls (18.0%). Patients in the 80+ age category had a higher likelihood of conveyance to hospital than patients in the 18–39 age category (OR2.53, p < 0.01). The likelihood of being conveyed to hospital rather than left at scene also increased with patient age (80+: OR3.12, p < 0.01; 18–39 OR1.26, p < 0.01). Patients not conveyed to hospital tended to be younger than those conveyed to hospital, mean age 56.4 vs. 62.4 years old. A total of 1.1% of patients’ age was unknown.

The ambulance service received most calls between 12:00 hrs and 18:00 hrs (n = 4019), with the lowest number of calls taking place between 00:00 hrs and 06:00 hrs (n = 1467). The data showed that 40.6% of calls occurred between 08:00 hrs and 18:00 hrs, Monday to Friday during GP working hours, with 59.4% calls occurring out-of-hours. People who made calls between 06:00 hrs and 18:00 hrs were significantly more likely to be conveyed to hospital than those who called between 18:00 hrs and 06:00 hrs (OR1.6, p < 0.01). There was a significant difference in the numbers of patients conveyed to hospital out-of-hours compared to during working hours (56.3% vs. 43.7% respectively, p < 0.01). However, patients were more likely to be conveyed to hospital than left at scene if they contacted the ambulance service during working hours compared to out-of-hours (OR2.13 vs. OR1.49, p < 0.01). Ambulance staff spent longer on scene during out-of-hours calls than working hours calls (r = 0.037, p = 0.002).

Most of the calls to the ambulance service originated from non-healthcare professionals (non-HCPs) (76.5%). However, of the calls made by HCPs, 90.6% of them were conveyed to hospital, compared to 54.8% of calls made from non-HCPs (OR8.0, p < 0.01). The ambulance service spent more time on scene at calls that originated from HCPs before conveying them to hospital (r = 0.083, p < 0.01).

Patients in the tenth decile of deprivation, the least deprived, were older than those in the first decile, the most deprived (61.53 years vs. 59.96 years). Calls to the NIAS were most frequent in the most deprived areas (15.3%), with the frequency decreasing as deprivation decreased (6.4%). Only 0.3% of calls were unable to be ranked.

The mean drive time was 17.8 minutes (SD = 11.2 minutes), with a maximum time recorded of 104.2 minutes. The mean distance from hospital was 15.6km (9.75 miles), with a maximum distance of 139.93km (87.46 miles). There were 36.9% of calls where the drive time and distance were not calculated as the patient was not conveyed to hospital. Of those that were calculated, the pick-up postcode was <15 minutes away from the hospital postcode in 49.8% of calls, with 86.4% of calls having a drive time of less than 30 minutes and only 0.4% calls having a drive time >60 minutes. The longer the drive time, the longer the time spent on scene before a patient was conveyed to hospital (r = 0.048, p < 0.01).

There were 38.0% of calls whereby the length of time spent at the scene by the ambulance was unknown, with 96.7% of these patients not conveyed to hospital. Of the 62% of times that were known, only 0.07% of patients were not conveyed to hospital, with the rest conveyed to hospital. Ambulance staff spent a mean length of time of 30.2 minutes on scene, with 46.3% of calls having ambulance staff on scene for 15–30 minutes. Only 14.5% of calls had an ambulance on scene for over 45 minutes. The length of time that the ambulance staff spent on scene correlates to patient age for patients that were conveyed to hospital (r = 0.974, p = 0.26), with more time spent on scene with older patients.

## Discussion

To the best of our knowledge, this research is the first to look at the characteristics of emergency calls to the ambulance service for diabetes-related problems in Northern Ireland. While some of the findings support previous research and findings from other areas, there are also several new findings presented.

### How do these results compare with what is already known?

Previous research has shown that patients who were not conveyed to hospital tended to be male and younger than those conveyed to hospital, with a mean age under 60 which is supported by this study. This is despite more calls relating to male patients, and is potentially due to the typecasting of older females as more vulnerable or to males refusing treatment and being unwilling to seek further care (Elwen et al., 2015; Villani et al., 2016). This could also be due to younger patients having fewer comorbidities and complex health issues and therefore being at lower risk of harm if not conveyed to hospital (Niroshan Siriwardena et al., 2019).

It has also been shown that when calls originated from HCPs, patients were significantly more likely to be conveyed to hospital than when calls originated from non-HCPs. This perhaps indicates that HCPs made more appropriate calls to the ambulance service based on their higher level of medical knowledge and expertise. It may also be due to ambulance staff being unwilling to question other HCPs out of deference to their professional status. Our results reflect previous research where calls made by HCPs were more likely to be conveyed to hospital despite accounting for fewer calls (O’Cathain et al., 2018).

This study also showed that patients were less likely to be conveyed to hospital out-of-hours, while day of the week did not influence conveyance rates. This was reflected in previous research showing that rates of conveyance differ across the day, with fewer patients conveyed to hospital at night time due to the reduced number of services available (Knowles et al., 2018; O’Cathain et al., 2018).

The results of this study reflect those of other studies that have shown that individuals in low socioeconomic areas seek more emergency care, resulting in higher volumes of ambulance calls (Noulas et al., 2018). This could be due to people from more deprived areas having poorer health literacy and less access to good quality medical care and being encouraged to contact emergency services, particularly out-of-hours (Peconi et al., 2017). Positive correlations have also been shown between deprivation, call rates and population density (Magnusson & Zelano, 2019). This is potentially due to more deprived areas having higher population density and so having more ambulance calls. However, Northern Ireland and Scotland do not have a high population density, suggesting that other factors are involved which require further investigation (Peacock & Peacock, 2006).

### What does this study add?

This study found that ambulance staff spent longer on scene during out-of-hours calls than during calls within working hours. This could potentially be due to decreased availability of services such as GP care, diabetic specialist nurses and telephone care outside working hours, leading ambulance staff to focus more on treating patients at home rather than conveying them to hospital (Knowles et al., 2018). It could also be in an attempt to cause less disruption to the patient by treating them on scene rather than conveying them to hospital during the night or out-of-hours.

The data also showed that for patients conveyed to hospital the longer the drive time to the hospital, the greater the length of time spent on scene. This was supported by previous research; however, the increased length of time on scene could not be attributed only to geographical location (Alanazy et al., 2019). This research also showed that paramedics spend longer on scene than EMTs and therefore it is a possibility that rural calls are attended to by paramedics who can provide greater treatment options and reduce the risk of conveyance, thus leading to more time spent on scene (Alanazy et al., 2019).

### Strengths and limitations

Although the data reflect a pre-COVID-19 pandemic timeframe, greater understanding of the use of ambulance services is now particularly pertinent as we know that adults with diabetes have been reluctant to attend hospitals for healthcare due to the greater fear of contracting the virus (Mansfield et al., 2021). Due to the large dataset and variety of demographics provided, these findings may be of relevance to other jurisdictions for people with diabetes and across other types of calls. However, there are a number of limitations associated with this research. Based on the data received, it was unclear which grade of ambulance staff attended the call and what treatment was provided. This did not allow for comparison of treatment and outcomes of patients with similar characteristics, based on whether they were attended to by a paramedic or an EMT. The dataset also contained missing data; however, as the data were missing completely at random and not systemically this did not lead to bias (Mack et al., 2018). The major limitation of this research was due to the absence of and lack of linkage of the clinical data and control centre data. Due to this, blood glucose levels were not available so calls could not be clinically categorised as either hypoglycaemic or hyperglycaemic. As the chief complaint was not assigned by the on-scene ambulance staff, not all patients on diabetes-related call-outs may have been captured and not all calls categorised as ‘Diabetes’ may have been related to diabetes. These limitations have shown the importance of integration of healthcare data systems to allow for improved access to and flow of data, more comprehensive research opportunities, better planning of services and improved care for patients (Coles, 2018).

## Conclusion

This research found that older males were more likely to contact the ambulance service but older females were more likely to be conveyed to hospital. The likelihood of conveyance increased if the call originated from an HCP or occurred during GP working hours. This research has shown the importance of data integration, as important questions such as the clinical reasoning for the call and how patient characteristics differed based on this could not be answered due to the lack of information available. Despite this, this study, as the first of its kind in Northern Ireland, plays an important role in the improvement of services within the NIAS. This study has also reinforced findings that people from more deprived areas and older individuals are more likely to contact the ambulance service and be conveyed to hospital, with individuals between 60 and 79 accounting for over a third of ambulance calls. Identifying the characteristics of individuals who contact the ambulance service and are conveyed to hospital for diabetes-related complications is of high importance so that alternative pathways of care or referral options can be devised to treat people closer to home or leave them safely at home. This has become particularly clear during the COVID-19 pandemic, as people have been reluctant to go to hospital for treatment.

## Author contributions

AW wrote the article, with VC, BCC and DMcC editing it. SM, NR and CC provided insights to the ambulance service and edited the article. BW provided support on the statistical analysis and edited the article. AW acts as the guarantor for this article.

## Conflict of interest

None declared.

## Ethics

Not required.

## Funding

This project is supported by the European Union’s INTERREG VA Programme, managed by the Special EU Programmes Board (SEUPB). The views and opinions expressed in this paper do not necessarily reflect the views of the European Commission or the Special EU Programmes Board (SEUPB).
